# SOCIAL STRESSORS ASSOCIATED WITH AGE-RELATED T LYMPHOCYTE PERCENTAGES IN OLDER US ADULTS: EVIDENCE FROM THE HRS

**DOI:** 10.1093/geroni/igac059.878

**Published:** 2022-12-20

**Authors:** Eric Klopack, Eileen Crimmins, Steve Cole, Teresa Seeman, Judith Carroll

**Affiliations:** University of Southern California, Leonard Davis School of Gerontology, Los Angeles, California, United States; University of Southern California, Los Angeles, California, United States; University of California Los Angeles, Los Angeles, California, United States; University of California Los Angeles, Los Angeles, California, United States; University of California, Los Angeles, Los Angeles, California, United States

## Abstract

Exposure to stress is a risk factor for poor health and accelerated aging. Immune aging, including declines in naïve and increases in late memory and terminally differentiated T cells, plays a role in immune health and tissue specific aging, and may contribute to elevated risk for poor health among those who experience high psychosocial stress. Past data have been limited in estimating the contribution of life stress to the development of accelerated immune aging and investigating mediators such as lifestyle and CMV infection. This study utilizes a national sample of 5744 US adults over age 50 to assess the relationship of social stress (viz., everyday discrimination, stressful life events, lifetime discrimination, life trauma, and chronic stress) with flow cytometric estimates of immune aging, including naïve and terminally differentiated T cell percentages and the ratio of CD4+ to CD8+ cells. Experiencing life trauma and chronic stress was related to a lower percentage of CD4+ naïve cells. Discrimination and chronic stress were each associated with a greater percentage of terminally differentiated CD4+ cells. Stressful life events, high lifetime discrimination, and life trauma were related to a lower percentage of CD8+ naïve cells. Stressful life events, high lifetime discrimination and chronic stress were associated with a higher percentage terminally differentiated CD8+ cells. High lifetime discrimination and chronic stress was related to a lower CD4+:CD8+ ratio. Lifestyle factors and CMV seropositivity partially reduced these effects. Results identify psychosocial stress as a contributor to accelerating immune aging by decreasing naïve and increasing senescent T cells.

